# Predicting mental health risk in adolescents using interpretable machine learning: Evidence from SHAP analysis

**DOI:** 10.1371/journal.pone.0359233

**Published:** 2026-10-21

**Authors:** Jinsheng He

**Affiliations:** College of Physical Education, Shanghai Normal University, Shanghai, China; Korea University - Seoul Campus: Korea University, KOREA, REPUBLIC OF

## Abstract

**Background/Objective:**

Adolescent mental health is a critical public health concern; however, the combined contribution of emotional, demographic, and behavioral factors to poor mental health remains incompletely understood. This study applied an interpretable machine learning framework to identify key predictors of elevated mental health risk among adolescents and characterize their relative and non-linear contributions.

**Methods:**

Data were drawn from the 2021 Youth Risk Behavior Survey (YRBS), comprising 16,946 U.S. high school students. Elevated mental health risk was defined using the YRBS current mental health item (Q85): reporting that mental health was not good most of the time or always during the past 30 days. The distinct 12-month sadness/hopelessness item (Q25) was retained as a predictor in the primary XGBoost model. The operating threshold was selected by maximizing the Youden index using five-fold out-of-fold predictions in the training set and was then locked before evaluation in the held-out test set. SHapley Additive exPlanations (SHAP) were used to assess feature importance and directional and non-linear patterns.

**Results:**

Overall, 28.0% of adolescents were classified as having elevated mental health risk, with higher prevalence among females (39.9% vs. 16.9%) and peak prevalence at ages 16–17. The primary XGBoost model achieved an AUC of 0.806 and PR-AUC of 0.603. At the training-derived threshold of 0.285, accuracy was 0.749, sensitivity 0.754, specificity 0.747, and balanced accuracy 0.750. SHAP analysis identified sadness/hopelessness as the dominant predictor, followed by sex, sleep duration, screen time, and breakfast frequency. In a sensitivity analysis excluding sadness/hopelessness, discrimination decreased (AUC 0.736; PR-AUC 0.510).

**Conclusion:**

The revised primary model achieved balanced sensitivity and specificity above 0.700 using a threshold selected entirely within the training data. Sadness/hopelessness was the strongest predictor, while demographic and behavioral factors provided additional predictive information. Because sadness/hopelessness is conceptually close to the current mental health outcome and the data are cross-sectional, the model should be interpreted as an auxiliary risk-stratification tool rather than a standalone clinical screening or diagnostic instrument.

## 1 Introduction

Mental health disorders are a leading cause of disability among adolescents worldwide. Epidemiological evidence indicates that approximately 10–20% of children and adolescents experience a diagnosable mental disorder annually, contributing substantially to years lived with disability and long-term functional impairment [1]. The onset of many internalizing disorders occurs during adolescence, which is a developmental stage characterized by rapid neurobiological and psychosocial change [2,3]. Heightened emotional reactivity, evolving peer relationships, and mounting academic pressures collectively increase vulnerability during this critical period [4,5]. Furthermore, consistent sex differences have been documented, with adolescent girls reporting higher levels of depressive symptoms and internalizing problems than their male counterparts [6,7]. Early identification of adolescents at elevated mental health risk is therefore essential for timely prevention and intervention.

Beyond emotional symptoms, lifestyle behaviors play an important role in shaping adolescent mental health. Sleep insufficiency has been associated with depressive symptoms and impaired emotional regulation [8–12]. Excessive screen time has been linked to lower psychological well-being, although the magnitude of this association varies across studies [13–15]. Physical activity, in contrast, is consistently associated with improved mood and reduced depressive symptoms [16–18]. Growing attention has also been directed toward dietary habits. Observational studies suggest that adherence to healthy dietary patterns, characterized by higher intake of fruits, vegetables, and whole foods, is associated with lower odds of depression and anxiety, whereas Western-style diets high in processed foods are linked to poorer mental health outcomes [19,20]. In adolescents specifically, dietary quality has been related to emotional well-being and depressive symptoms independently of socioeconomic factors [21]. These findings support the notion that nutrition may influence mental health through inflammatory pathways, oxidative stress, and gut–brain interactions. Importantly, sleep, sedentary behavior, physical activity, and diet coexist within a 24-hour behavioral framework, suggesting that mental health risk may arise from the cumulative influence of multiple modifiable behaviors rather than isolated exposures [22]. However, most prior studies have relied on conventional linear regression models and have rarely examined non-linear gradients or interaction structures among these behaviors.

Machine learning methods offer a compelling approach to address these limitations. In mental health research, machine learning models have been applied to predict suicidal attempts [23,24] and adolescent subjective well-being [25]. Tree-based ensemble methods, such as gradient boosting, can capture non-linear associations and higher-order interactions without imposing strict parametric assumptions [26]. Nevertheless, interpretability remains a central challenge, particularly in high-stakes domains such as mental health screening [27]. The SHapley Additive exPlanations (SHAP) framework addresses this limitation by quantifying each predictor's contribution to model output based on game-theoretic principles [28]. SHAP enables both global feature ranking and individual-level effect visualization, thereby enhancing the interpretability and potential clinical utility of machine learning models.

Despite growing interest in machine learning for adolescent mental health, few studies have applied SHAP-based approaches to a large national YRBS sample while jointly examining emotional, demographic, and behavioral predictors and explicitly evaluating how model performance changes when a proximal emotional indicator is removed. The present study used the 2021 YRBS to develop an interpretable gradient boosting model for elevated current mental health risk. Specifically, the objectives were to evaluate predictive performance using a training-derived classification threshold, quantify the relative importance and non-linear patterns of predictors using SHAP, examine robustness to exclusion of the distinct 12-month sadness/hopelessness item, compare XGBoost with simpler benchmark models, and assess portability across demographic groups. This approach was intended to provide a transparent assessment of predictive performance and the contribution of modifiable and non-modifiable factors to adolescent mental health risk stratification.

## 2 Method

### 2.1 Data source and study population

Data for this study were derived from the Youth Risk Behavior Survey (YRBS), a nationally representative survey conducted biennially by the Centers for Disease Control and Prevention (CDC) to monitor health-risk behaviors among U.S. high school students [29]. The YRBS employs a three-stage cluster sampling design to obtain representative samples of students in grades 9–12 across the United States. Specifically, large counties or groups of smaller counties are selected at the first stage; schools within these counties are sampled at the second stage; and intact classrooms within selected schools are randomly chosen at the third stage [30,31]. The dataset was accessed for research purposes on 08 December 2025. The authors did not have access to information that could identify individual participants during or after data collection.

The present study conducted a secondary cross-sectional analysis of the 2021 YRBS public-use dataset. The analytic sample was restricted to participants with complete data on the outcome and all predictors of interest; those with missing or invalid responses on key study variables were excluded. The final analytic sample comprised 16,946 adolescents, as detailed in the Results section. The reduced reproducible analytic dataset used for the present modeling did not retain the YRBS survey weight, stratum, or PSU variables; therefore, descriptive percentages reported in this manuscript represent unweighted proportions of the analytic sample rather than design-weighted national prevalence estimates. Given that this analysis relied exclusively on publicly available, de-identified data, it was exempt from institutional review board approval [32]. All relevant data are within the manuscript and S1 Data.

### 2.2 Measures: Outcome and predictor variables

The primary outcome was a binary indicator of elevated current mental health risk based on YRBS Q85. Participants were asked, “During the past 30 days, how often was your mental health not good? (Poor mental health includes stress, anxiety, and depression.)” Response options were never, rarely, sometimes, most of the time, and always. Consistent with the YRBS dichotomous definition, participants who answered “most of the time” or “always” were classified as elevated risk (coded 1), while those who answered “never,” “rarely,” or “sometimes” were classified as non-risk (coded 0) [33].

Predictors were organized into emotional, demographic, and behavioral/lifestyle domains. The emotional predictor was the distinct YRBS Q25 sadness/hopelessness item, which asked whether, during the past 12 months, participants had felt so sad or hopeless almost every day for two weeks or more in a row that they stopped doing some usual activities (yes/no) [33]. Demographic predictors included age (≤12, 13, 14, 15, 16, 17, and ≥18 years), sex (female or male), and race/ethnicity (White, Black or African American, Hispanic/Latino, or Other). Behavioral and lifestyle factors included sleep duration, screen time, moderate-to-vigorous physical activity (MVPA) days, breakfast frequency, fruit consumption, green salad intake, potato consumption, and carrot intake. Body weight perception was additionally included as a self-reported measure.

Missing data were handled using a complete-case approach. Age, sex, race/ethnicity, body weight perception, and sadness/hopelessness were treated as categorical variables and one-hot encoded before model fitting. The remaining behavioral variables were retained in their analytic numeric coding. As tree-based models such as XGBoost do not require feature scaling [34], no standardization was applied for XGBoost modeling. Approximately 28% of the analytic sample met the elevated-risk definition, and class imbalance was addressed through multi-metric evaluation, threshold calibration, and sensitivity analyses [35].

### 2.3 Primary predictor set and no-sadness sensitivity analysis

The primary predictor set included the 12-month sadness/hopelessness indicator together with all demographic and behavioral/lifestyle predictors. Q25 sadness/hopelessness and Q85 current mental health are distinct YRBS items with different wording and reference periods; therefore, Q25 does not directly define the outcome. However, because sadness/hopelessness is conceptually proximal to current poor mental health and may overlap temporally with the 30-day outcome window, we additionally fitted a no-Sadness XGBoost model as a sensitivity analysis. This analysis was used to quantify how strongly model discrimination and case detection depended on the proximal emotional predictor.

### 2.4 Machine learning model development and evaluation

The XGBoost algorithm was selected as the primary modeling approach because it can model non-linear relationships in structured tabular data and can be interpreted using SHAP [32,36]. The dataset was partitioned into training (70%) and held-out test (30%) sets using stratified sampling by outcome. Within the training set, five-fold cross-validation was stratified by mental health risk status, sex, and age group. Hyperparameters were tuned using a Cartesian grid comprising eta (0.05, 0.15), max_depth (2, 4), min_child_weight (1), subsample (0.8), colsample_bytree (0.8), and scale_pos_weight (1 or the training-set non-risk:risk ratio, approximately 2.58). The maximum number of boosting rounds was 250, AUC was the evaluation metric, and early stopping was applied after 15 rounds without improvement. The selected primary model used eta = 0.05, max_depth = 2, min_child_weight = 1, subsample = 0.8, colsample_bytree = 0.8, scale_pos_weight = 1, and 227 boosting rounds, with a cross-validated AUC of 0.812. To determine an operating threshold without using the test data, out-of-fold probabilities were generated for the training sample using the selected model specification. The threshold that maximized the Youden index (sensitivity + specificity − 1) was 0.285 and was locked before evaluation on the held-out test set. Final performance was assessed using accuracy, sensitivity, specificity, positive predictive value (PPV), negative predictive value (NPV), F1-score, balanced accuracy, AUC, and PR-AUC. Additional sensitivity analyses included a downsampled XGBoost model, a numeric SMOTE-based XGBoost model, and the no-Sadness model. The downsampled and SMOTE models retained sadness/hopelessness and were evaluated at the default threshold of 0.50. A White-only internal validation was also conducted using the primary predictor set, a 70/30 split, and five-fold training cross-validation stratified by outcome, sex, and age group. Logistic regression, an R `lm` linear probability model, and random forest were fitted using the same primary predictor set as benchmark models and evaluated on the same held-out test set at threshold 0.50. Finally, cross-demographic transfer validation trained and tested the primary predictor specification across White/non-White and female/male groups; these transfer analyses used the default threshold of 0.50.

### 2.5 Model interpretation, PCA diagnostics, and statistical analysis

Model interpretability was assessed using SHapley Additive exPlanations (SHAP) [28]. Global feature importance was quantified using mean absolute SHAP values. SHAP summary (beeswarm) plots were used to display the distribution and direction of feature contributions across participants, and dependence plots were generated for the highest-ranked predictors to examine non-linear predictor effects across their observed values. Because categorical variables were one-hot encoded, SHAP outputs for these variables are displayed as level-specific indicator features.

To explore unsupervised structure in the complete primary predictor set, principal component analysis (PCA) was applied to the standardized model matrix including sadness/hopelessness. The first two principal components were plotted and colored by elevated mental health risk status. Additional exploratory PCA plots colored by sex and age group were generated to assess whether visible structure was primarily associated with demographic grouping. The proportion of variance explained by each principal component was reported.

Descriptive analyses were used to characterize the complete-case analytic sample. Counts and unweighted percentages were calculated for demographic characteristics by outcome status, and between-group differences were assessed using chi-square tests for categorical variables, with statistical significance defined as p < 0.05. Machine learning modeling, sensitivity analyses, PCA diagnostics, SHAP-based interpretation, figure generation, and result export were implemented in R. The complete reproducible R code is provided in S1 File.

## 3 Results

### 3.1 Study population characteristics

A total of 16,946 adolescents were included in the analysis, of whom 4,740 (28.0%) were classified as having elevated mental health risk and 12,206 (72.0%) as non-risk. Table 1 summarizes sample characteristics by outcome status. All demographic variables were significantly associated with elevated risk (all p < 0.001).

**Table 1:** Demographic Characteristics of the Study Participants by Outcome Status.

variables	0	percent	1	percent	all	percent	P
age							<0.001
12 y or younger	23	67.65%	11	32.35%	34	0.20%	
13 y	47	78.33%	13	21.67%	60	0.35%	
14 y	2513	74.97%	839	25.03%	3352	19.78%	
15 y	3142	71.80%	1234	28.20%	4376	25.82%	
16 y	2978	70.30%	1258	29.70%	4236	25.00%	
17 y	2746	70.88%	1128	29.12%	3874	22.86%	
18 y	757	74.65%	257	25.35%	1014	5.98%	
sex							<0.001
Female	4897	60.12%	3248	39.88%	8145	48.06%	
Male	7309	83.05%	1492	16.95%	8801	51.94%	
race							<0.001
White	6589	71.46%	2632	28.54%	9221	54.41%	
Black or African American	1777	75.55%	575	24.45%	2352	13.88%	
Hispanic/Latino	2360	72.46%	897	27.54%	3257	19.22%	
All Other Races	1480	69.94%	636	30.06%	2116	12.49%	
Body weight							<0.001
Underweight	394	69.61%	172	30.39%	566	3.34%	
Normal weight	7852	73.37%	2850	26.63%	10702	63.15%	
Overweight	1912	69.78%	828	30.22%	2740	16.17%	
Obesity	2048	69.71%	890	30.29%	2938	17.34%	
Sum	12206		4740		16946		

The prevalence of elevated risk followed a discernible age-related pattern, rising from early to mid-adolescence. Prevalence was highest at ages 16 and 17 (29.7% and 29.1%, respectively), compared with lower proportions at younger ages (25.0% at age 14 and 28.2% at age 15), and declined modestly to 25.4% at age 18. Adolescents aged 12 years or younger and those aged 13 represented a very small fraction of the total sample.

Sex differences were particularly pronounced. Elevated risk was nearly 2.4 times more prevalent among females (39.9%) than males (17.0%), representing the largest demographic disparity observed across all variables examined. With respect to race and ethnicity, the highest prevalence was recorded in the Other racial/ethnic group (30.1%), followed by White (28.5%) and Hispanic/Latino (27.5%) adolescents, while Black adolescents had a comparatively lower prevalence (24.5%). Although statistically significant, these racial and ethnic differences were modest in magnitude.

Body weight perception also exhibited a discernible gradient. Adolescents who perceived themselves as underweight, obese, or overweight had broadly similar and elevated prevalences (30.4%, 30.3%, and 30.2%, respectively), whereas those with a normal weight perception had the lowest prevalence (26.6%). Overall, sex and age were the most consequential demographic correlates of elevated mental health risk, while differences across racial/ethnic and weight perception categories, though significant, were comparatively small.

### 3.2 Predictive performance of the primary XGBoost model

Table 2 presents the performance of the primary XGBoost model on the held-out test set. At the default threshold of 0.50, the model achieved an accuracy of 0.783, sensitivity of 0.534, specificity of 0.879, balanced accuracy of 0.707, AUC of 0.806, and PR-AUC of 0.603. The classification threshold was then selected entirely within the training data using five-fold out-of-fold predictions and the Youden index. The resulting threshold of 0.285 was locked before test-set evaluation. At this threshold, the model achieved an accuracy of 0.749, sensitivity of 0.754, specificity of 0.747, PPV of 0.536, NPV of 0.886, F1-score of 0.627, balanced accuracy of 0.750, AUC of 0.806, and PR-AUC of 0.603.

**Table 2:** Predictive Performance of the Primary XGBoost Model at the Training-Derived Threshold.

Metric	Value
Test set, n	5,083
At-risk cases, n	1,422
Non-risk cases, n	3,661
Threshold	0.285
Accuracy	0.749
Sensitivity	0.754
Specificity	0.747
Positive predictive value	0.536
Negative predictive value	0.886
F1-score	0.627
Balanced accuracy	0.750
AUC	0.806
PR-AUC	0.603
True positives	1,072
True negatives	2,733
False positives	928
False negatives	350

Note. The primary XGBoost model included sadness/hopelessness. The operating threshold (0.285) was selected by maximizing the Youden index using five-fold out-of-fold predictions within the training set and was locked before evaluation on the held-out test set. AUC, area under the receiver operating characteristic curve; PR-AUC, area under the precision–recall curve.

The training-derived threshold therefore produced a substantially more balanced operating profile than the default 0.50 threshold: sensitivity increased from 0.534 to 0.754 while specificity remained 0.747. Both sensitivity and specificity exceeded 0.700 in the held-out test set. The unchanged AUC and PR-AUC reflect that threshold calibration altered classification performance rather than the underlying ranking ability of the model.

### 3.3 Threshold calibration and class-imbalance sensitivity analyses

Sensitivity analyses supported the robustness of the balanced performance profile (Table 3). At the default threshold of 0.50, downsampling produced an accuracy of 0.744, sensitivity of 0.761, specificity of 0.737, balanced accuracy of 0.749, AUC of 0.804, and PR-AUC of 0.598. The SMOTE-based model achieved an accuracy of 0.752, sensitivity of 0.719, specificity of 0.765, balanced accuracy of 0.742, AUC of 0.792, and PR-AUC of 0.582.

**Table 3:** Threshold, Class-Imbalance, and Predictor-Set Sensitivity Analyses of the Primary XGBoost Model.

Model	Accuracy	Sensitivity	Specificity	PPV	NPV	F1-score	Balanced accuracy	AUC	PR-AUC
Primary XGBoost, threshold 0.50	0.783	0.534	0.879	0.632	0.829	0.579	0.707	0.806	0.603
Primary XGBoost, CV-locked threshold 0.285	0.749	0.754	0.747	0.536	0.886	0.627	0.750	0.806	0.603
Downsampled XGBoost, threshold 0.50	0.744	0.761	0.737	0.529	0.888	0.624	0.749	0.804	0.598
SMOTE-based XGBoost, threshold 0.50	0.752	0.719	0.765	0.543	0.875	0.618	0.742	0.792	0.582
No-Sadness XGBoost sensitivity analysis, threshold 0.50	0.742	0.238	0.939	0.600	0.760	0.341	0.588	0.736	0.510

Note. The primary model included sadness/hopelessness. The CV-selected threshold of 0.285 was determined using five-fold out-of-fold predictions in the training data and locked before test-set evaluation. Downsampled, SMOTE-based, and no-Sadness sensitivity models were evaluated at the default threshold of 0.50. PPV, positive predictive value; NPV, negative predictive value; AUC, area under the receiver operating characteristic curve; PR-AUC, area under the precision–recall curve.

These results indicate that balanced case detection was not unique to the threshold-calibrated primary model. Both resampling approaches also achieved sensitivity and specificity above 0.700, although their AUC and PR-AUC did not exceed those of the primary XGBoost model. The training-derived threshold was therefore retained as the main operating threshold because it improved sensitivity-specificity balance without altering the training sample through resampling.

The primary model at threshold 0.285 yielded 1,072 true positives, 2,733 true negatives, 928 false positives, and 350 false negatives in the held-out test set. Compared with the default 0.50 threshold, this substantially reduced false negatives while accepting a higher number of false positives, a trade-off that is relevant when the intended use emphasizes identification of adolescents who may benefit from further assessment.

To examine robustness within a more homogeneous racial/ethnic subgroup, a White-only internal validation was conducted using the primary predictor set including sadness/hopelessness. The White-only sample was split into training and test sets, with five-fold cross-validation within training stratified by mental health risk status, sex, and age group.

In the White-only held-out test set (n = 2,765; 789 elevated-risk and 1,976 non-risk participants), the model achieved an accuracy of 0.770, sensitivity of 0.757, specificity of 0.775, PPV of 0.573, NPV of 0.889, F1-score of 0.652, balanced accuracy of 0.766, AUC of 0.825, and PR-AUC of 0.650. Thus, sensitivity and specificity also exceeded 0.700 within this subgroup at the default threshold of 0.50.

### 3.4 No-sadness sensitivity analysis

Because the 12-month sadness/hopelessness item is conceptually proximal to the current mental health outcome, we fitted a separate XGBoost model excluding this predictor. At the default threshold of 0.50, the no-Sadness model achieved an accuracy of 0.742, sensitivity of 0.238, specificity of 0.939, balanced accuracy of 0.588, AUC of 0.736, and PR-AUC of 0.510. By comparison, the primary model including sadness/hopelessness at the same 0.50 threshold achieved an AUC of 0.806, PR-AUC of 0.603, sensitivity of 0.534, and balanced accuracy of 0.707.

The reduction in AUC, PR-AUC, sensitivity, and balanced accuracy after excluding sadness/hopelessness indicates that this distinct emotional indicator contributed substantial predictive information. At the same time, the no-Sadness model retained moderate discrimination, showing that demographic and behavioral predictors continued to provide meaningful information when the proximal emotional predictor was removed.

### 3.5 Benchmark comparison with simpler models

To evaluate whether XGBoost provided additional predictive value beyond simpler approaches, we compared the primary XGBoost model with logistic regression, a linear probability model, and random forest using the same predictor set including sadness/hopelessness and the same held-out test set. For a common threshold-based comparison, all models in Table 4 were evaluated at the default threshold of 0.50. XGBoost achieved an AUC of 0.806 and PR-AUC of 0.603. Logistic regression achieved an AUC of 0.805 and PR-AUC of 0.602, the linear probability model achieved an AUC of 0.805 and PR-AUC of 0.602, and random forest achieved an AUC of 0.793 and PR-AUC of 0.580.

**Table 4:** Benchmark Comparison of the Primary XGBoost Model with Simpler Models.

Model	Accuracy	Sensitivity	Specificity	PPV	NPV	F1-score	Balanced accuracy	AUC	PR-AUC
XGBoost	0.783	0.534	0.879	0.632	0.829	0.579	0.707	0.806	0.603
Logistic regression	0.778	0.557	0.864	0.614	0.834	0.584	0.711	0.805	0.602
Linear probability model	0.778	0.598	0.848	0.605	0.845	0.602	0.723	0.805	0.602
Random forest	0.777	0.511	0.880	0.623	0.822	0.561	0.695	0.793	0.580

Note. All models used the same predictor set including sadness/hopelessness and were evaluated on the same held-out test set at the default probability threshold of 0.50 to enable a common threshold-based comparison. PPV, positive predictive value; NPV, negative predictive value; AUC, area under the receiver operating characteristic curve; PR-AUC, area under the precision–recall curve.

Discrimination was therefore very similar for XGBoost, logistic regression, and the linear probability model, while random forest was modestly lower. The added value of XGBoost in this study lies primarily in its flexible modeling and SHAP-based visualization of feature contributions and non-linear patterns rather than a large gain in AUC over simpler models.

In the logistic regression benchmark, not reporting the 12-month sadness/hopelessness experience was associated with markedly lower odds of elevated current mental health risk (OR = 0.150, 95% CI: 0.136–0.166, p < 0.001). Male sex was also associated with lower odds compared with female sex (OR = 0.469, 95% CI: 0.425–0.518, p < 0.001). Higher screen time was associated with higher odds of elevated risk (OR = 1.156, 95% CI: 1.119–1.195, p < 0.001), whereas longer sleep duration (OR = 0.826, 95% CI: 0.797–0.856, p < 0.001), higher breakfast frequency (OR = 0.956, 95% CI: 0.938–0.975, p < 0.001), and more MVPA days (OR = 0.975, 95% CI: 0.956–0.995, p = 0.014) were associated with lower odds. These findings were broadly consistent with the direction of the SHAP-based patterns.

### 3.6 Global feature importance in the primary model

Fig 1 displays global feature importance ranked by mean absolute SHAP values in the primary XGBoost model. The one-hot indicator corresponding to the sadness/hopelessness variable showed by far the largest contribution (mean absolute SHAP = 0.955), followed by sex (0.373), sleep duration (0.198), screen time (0.165), and breakfast frequency (0.099). Race/ethnicity indicator levels, body weight perception, potato consumption, MVPA days, and age indicators contributed smaller amounts.

**Fig 1:**
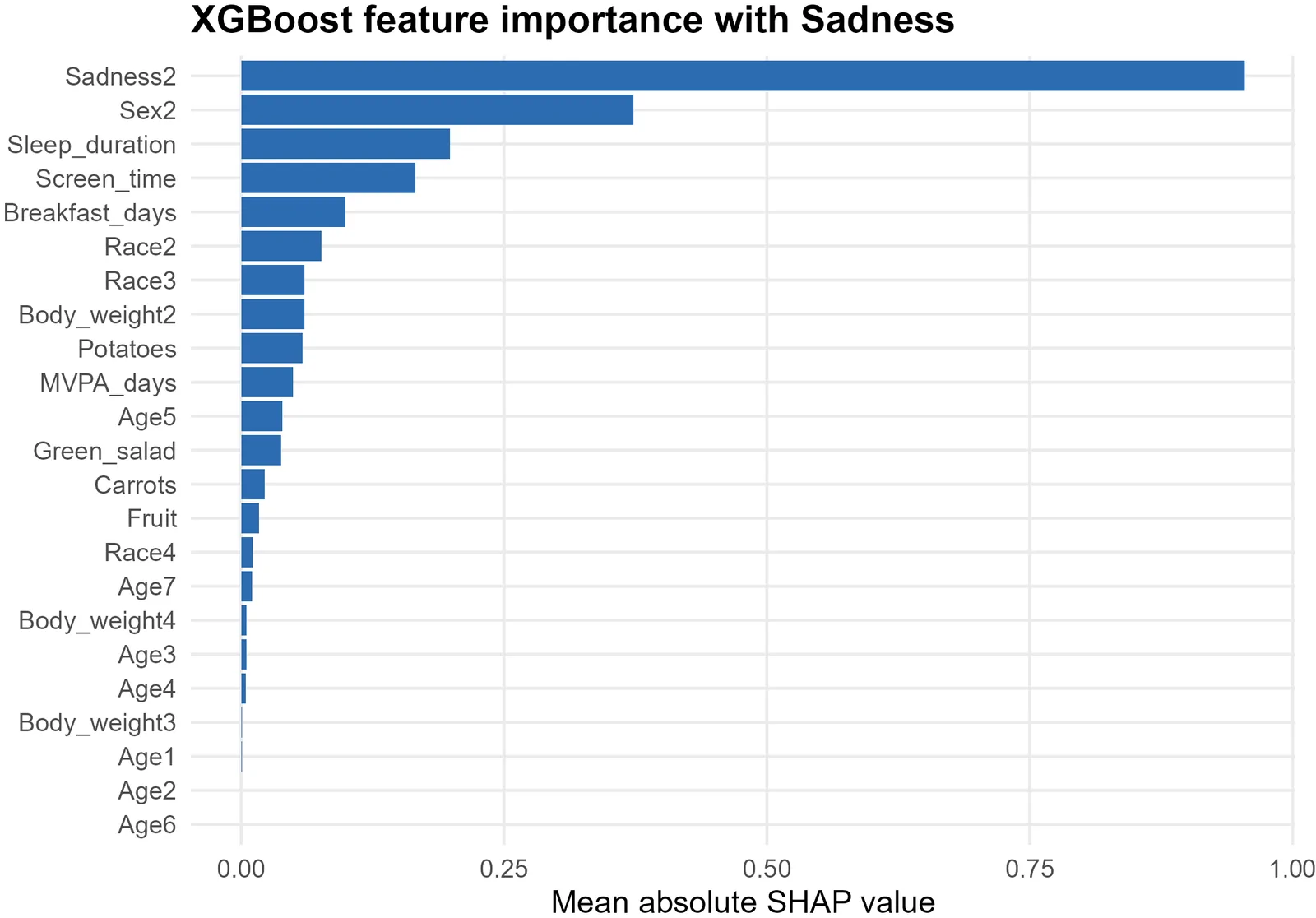
Global feature importance based on mean absolute SHAP values in the primary XGBoost model including sadness/hopelessness.

The dominant contribution of sadness/hopelessness is consistent with its conceptual proximity to current poor mental health, while the remaining SHAP pattern indicates that demographic and behavioral factors added predictive information beyond this emotional indicator. SHAP values reflect contributions to model prediction and should not be interpreted as causal effects.

### 3.7 Directional patterns and interpretable predictor effects in the primary model

#### 3.7.1 Distribution and directionality of SHAP effects.

Fig 2 presents the SHAP summary (beeswarm) plot for the primary XGBoost model. Each point represents an individual observation and the x-axis denotes the corresponding SHAP value. Categorical predictors were one-hot encoded, so level-specific indicators such as Sadness2 and Sex2 appear as separate model features.

**Fig 2:**
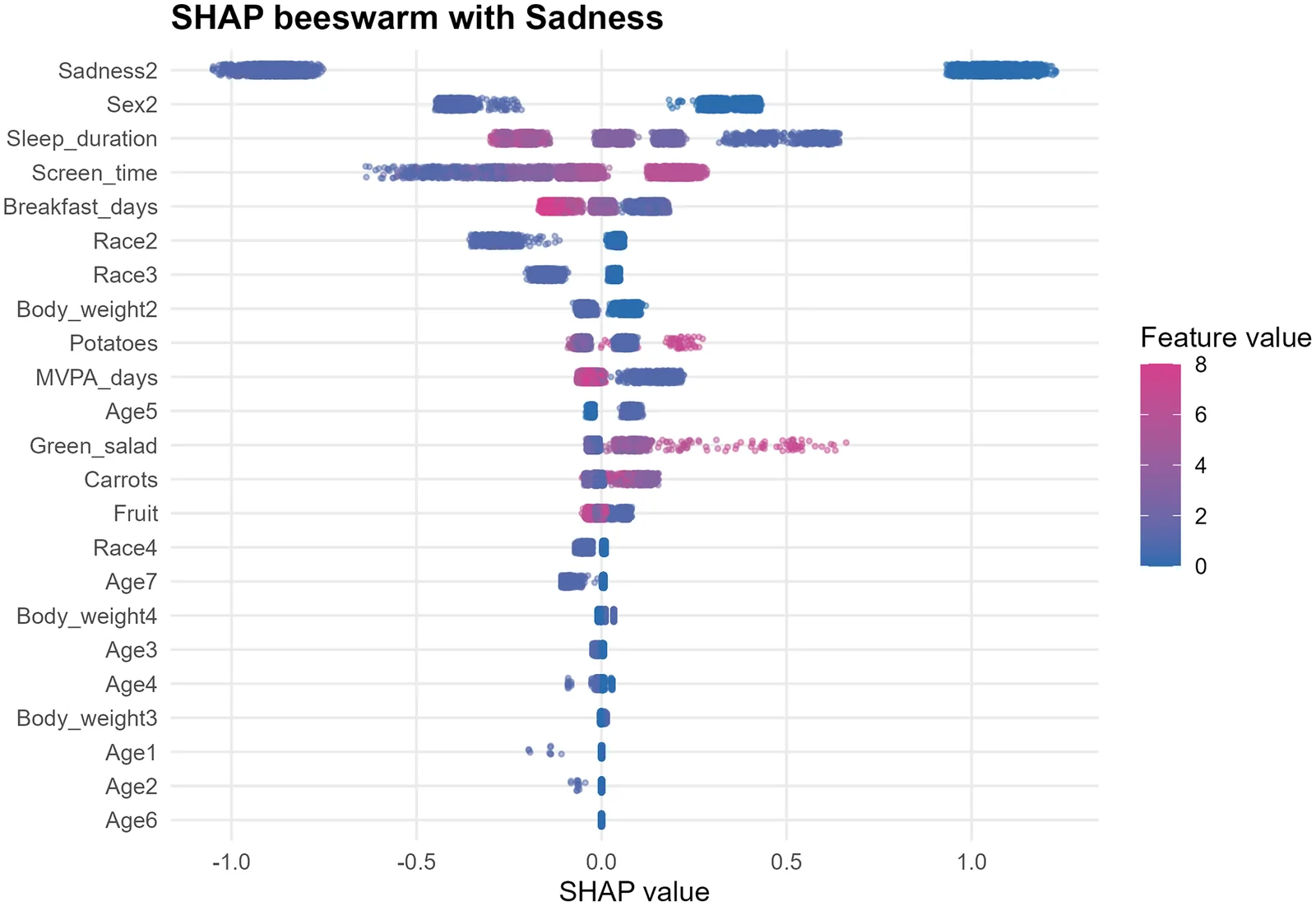
SHAP beeswarm plot showing the distribution and directionality of predictors in the primary XGBoost model. Categorical variables were one-hot encoded; positive SHAP values indicate contributions toward higher predicted risk and negative values indicate contributions toward lower predicted risk.

The sadness/hopelessness indicator showed the clearest separation in SHAP values. The indicator corresponding to not reporting sadness/hopelessness was associated with a large negative contribution to predicted risk, whereas reporting the sadness/hopelessness experience corresponded to substantially higher predicted risk. Male sex also generally contributed toward lower predicted risk relative to female sex. Longer sleep duration tended to align with lower predicted risk, while higher screen time contributed toward higher predicted risk. Breakfast frequency and MVPA days showed smaller generally protective patterns.

#### 3.7.2 Predictor distributions by outcome status.

To improve the readability of key patterns, Fig 3 presents violin and box plots for sadness/hopelessness, sleep duration, screen time, and MVPA days by elevated mental health risk status. Participants classified as elevated risk were more likely to report the 12-month sadness/hopelessness experience and generally showed shorter sleep duration, higher screen time, and lower physical activity. These distributional patterns were broadly consistent with the SHAP-based interpretation.

**Fig 3:**
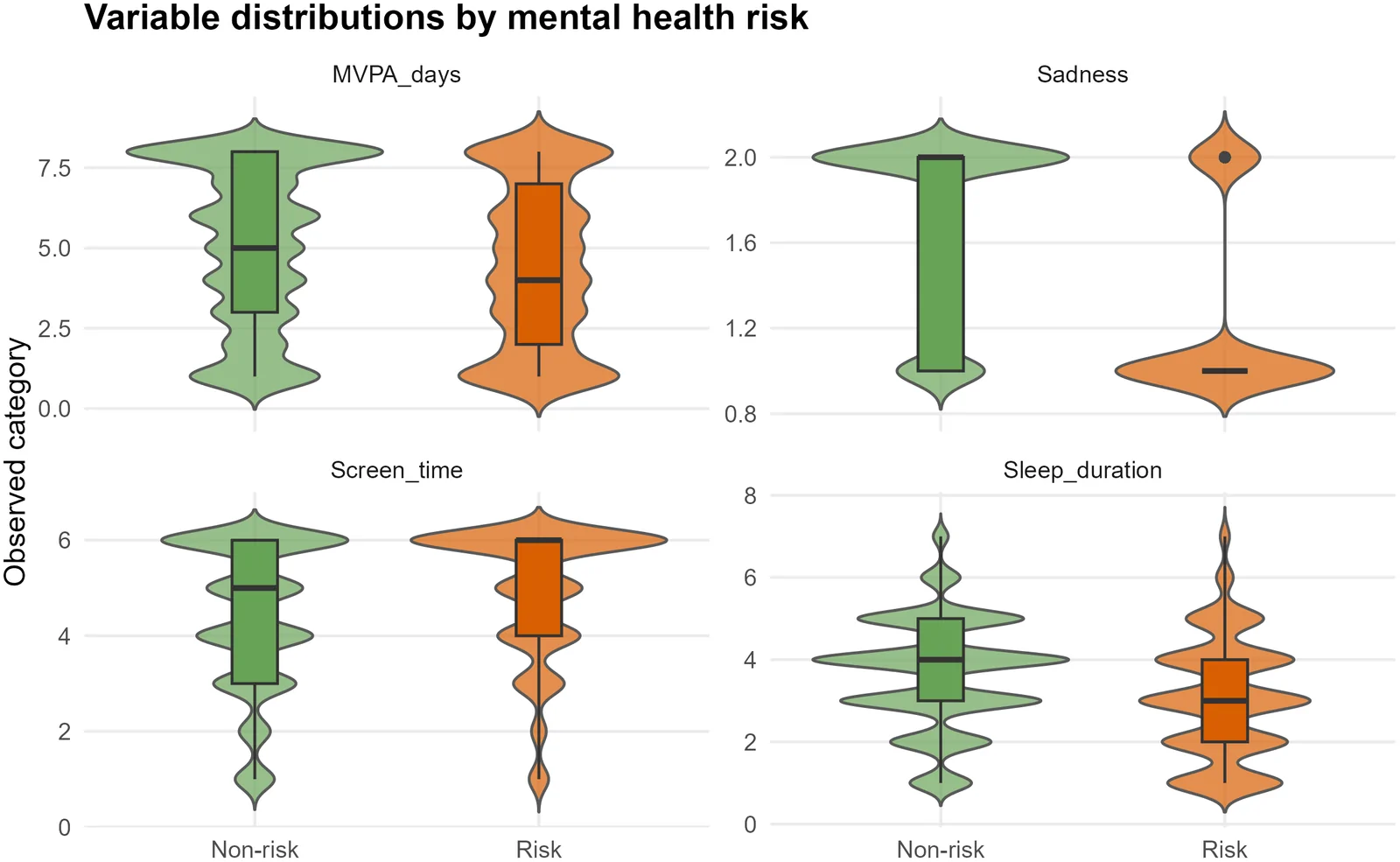
Distributions of selected predictors by elevated mental health risk status. Violin and box plots show sadness/hopelessness, sleep duration, screen time, and MVPA days in the non-risk and elevated-risk groups.

#### 3.7.3 Dependence patterns among top predictors.

SHAP dependence plots for the top-ranked predictors were retained as S1 Fig to provide additional information on the relationship between observed predictor levels and their SHAP contributions.

#### 3.7.4 Summary of interpretable patterns.

Taken together, the SHAP beeswarm, dependence plots, and predictor distribution plots indicate that the primary model relied strongly on the 12-month sadness/hopelessness indicator, with additional contributions from sex, sleep duration, screen time, breakfast frequency, race/ethnicity, physical activity, and other measured factors. The results therefore suggest a combination of proximal emotional status, demographic characteristics, and health behaviors rather than a purely behavioral risk profile.

### 3.8 Unsupervised PCA analysis of the primary predictor set

To examine whether the complete primary predictor set showed clear intrinsic structure by outcome status, PCA was performed using the standardized model matrix including sadness/hopelessness. The first principal component explained 10.48% of the variance and the second explained 7.81%, together accounting for 18.28% of total variance.

Fig 4 shows substantial overlap between adolescents with and without elevated mental health risk in the first two principal components. Thus, despite the strong predictive importance of sadness/hopelessness in the supervised model, the complete predictor set did not produce a simple two-dimensional separation by outcome status. Additional PCA plots colored by sex and age group are provided as S2 and S3 Figs and likewise showed substantial overlap across groups.

**Fig 4:**
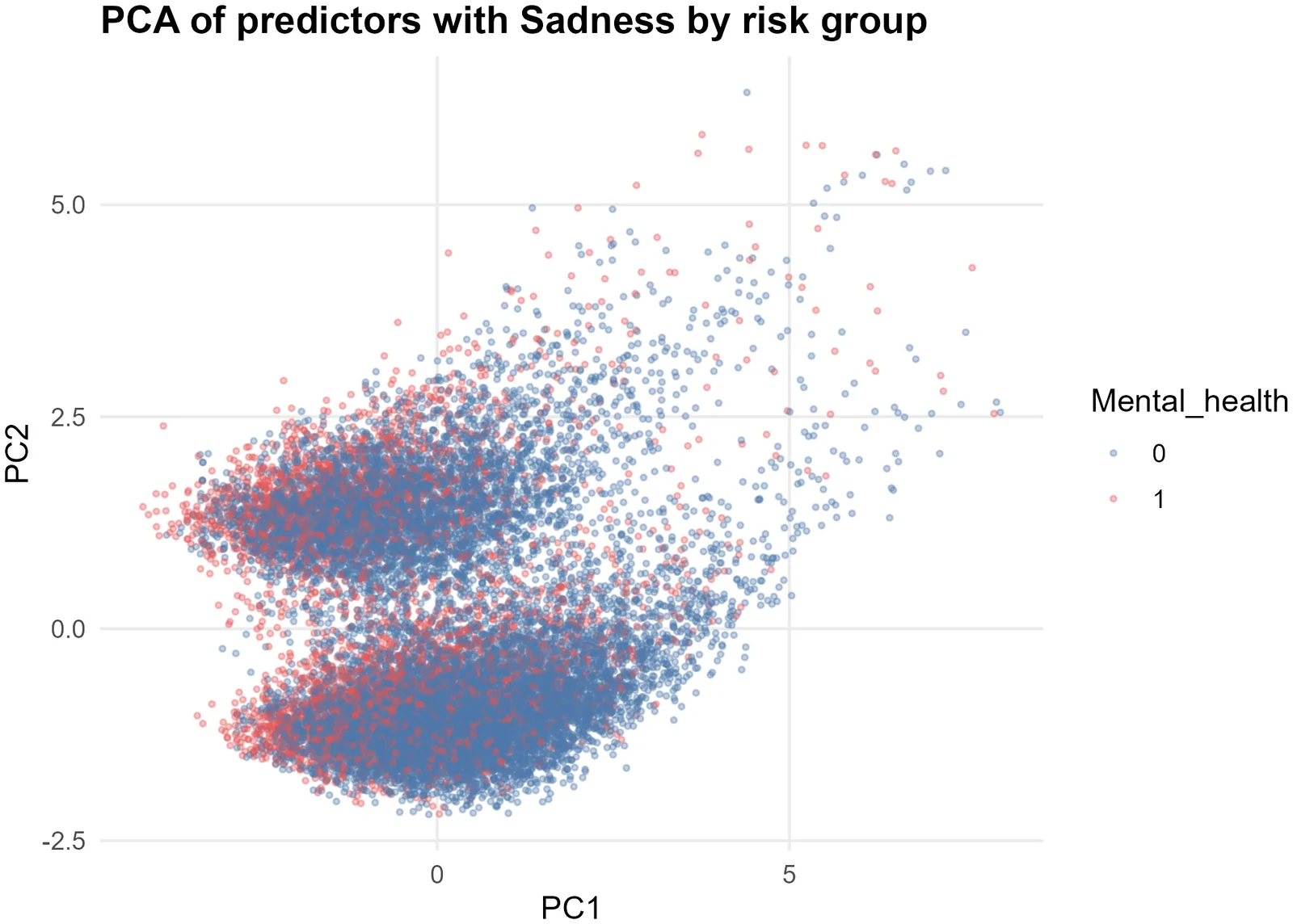
Principal component analysis of the primary predictor set, including sadness/hopelessness, by elevated mental health risk status. Points represent individual participants projected onto the first two principal components; substantial overlap indicates the absence of clear two-dimensional unsupervised separation.

### 3.9 Cross-demographic transfer validation

To evaluate portability of the primary model across demographic groups, cross-demographic transfer validation was conducted by training the same predictor specification in one subgroup and evaluating it in another (Table 5). All transfer analyses included sadness/hopelessness and used the default threshold of 0.50.

**Table 5:** Cross-Demographic Transfer Validation of the Primary XGBoost Model.

Training group	Testing group	n	At-risk, n	Non-risk, n	Accuracy	Sensitivity	Specificity	PPV	NPV	F1-score	Balanced accuracy	AUC	PR-AUC
White	non-White	7,725	2,108	5,617	0.770	0.618	0.828	0.574	0.852	0.595	0.723	0.800	0.583
non-White	White	9,221	2,632	6,589	0.775	0.393	0.927	0.683	0.793	0.499	0.660	0.815	0.628
Female	Male	8,801	1,492	7,309	0.789	0.653	0.817	0.421	0.920	0.512	0.735	0.777	0.461
Male	Female	8,145	3,248	4,897	0.706	0.800	0.643	0.598	0.829	0.684	0.722	0.768	0.659

Note. All transfer-validation models included sadness/hopelessness and used the same primary predictor specification. Models were trained in one demographic subgroup and evaluated in another at the default probability threshold of 0.50. PPV, positive predictive value; NPV, negative predictive value; AUC, area under the receiver operating characteristic curve; PR-AUC, area under the precision–recall curve.

When trained on White participants and tested on non-White participants, the model achieved an accuracy of 0.770, sensitivity of 0.618, specificity of 0.828, balanced accuracy of 0.723, AUC of 0.800, and PR-AUC of 0.583. In the reverse direction, training on non-White participants and testing on White participants yielded an accuracy of 0.775, sensitivity of 0.393, specificity of 0.927, balanced accuracy of 0.660, AUC of 0.815, and PR-AUC of 0.628.

Sex-based transfer validation also showed threshold-dependent imbalance. Training on female participants and testing on male participants yielded an accuracy of 0.789, sensitivity of 0.653, specificity of 0.817, balanced accuracy of 0.735, AUC of 0.777, and PR-AUC of 0.461. Training on male participants and testing on female participants yielded an accuracy of 0.706, sensitivity of 0.800, specificity of 0.643, balanced accuracy of 0.722, AUC of 0.768, and PR-AUC of 0.659.

These results indicate that rank discrimination remained moderate to good across transfer settings, but sensitivity and specificity varied substantially by training-testing direction. The most pronounced imbalance occurred when the model was trained on non-White participants and tested on White participants, where sensitivity was 0.393 despite specificity of 0.927. This pattern supports subgroup-specific threshold calibration and external validation before practical use.

## 4 Discussion

This study applied an interpretable machine learning framework to classify elevated current mental health risk among adolescents using a combination of emotional, demographic, and behavioral predictors. After correcting the predictor specification to distinguish the 12-month sadness/hopelessness item (Q25) from the current mental health outcome (Q85), the primary XGBoost model achieved an AUC of 0.806. A threshold selected exclusively from training out-of-fold predictions produced sensitivity of 0.754 and specificity of 0.747 in the held-out test set. Sadness/hopelessness was the dominant SHAP predictor, followed by sex, sleep duration, screen time, and breakfast frequency. A no-Sadness sensitivity analysis showed substantially lower discrimination and case detection, while benchmark and subgroup analyses provided additional context regarding model robustness and portability.

### 4.1 Principal findings

#### 4.1.1 Overall risk prevalence and developmental pattern.

Overall, 28.0% of adolescents were classified as having elevated mental health risk, a proportion that highlights the substantial public health burden of adolescent psychological distress. The prevalence of elevated risk increased during mid-adolescence and peaked at ages 16–17. This developmental pattern is consistent with prior evidence showing that internalizing symptoms and psychological vulnerability often intensify during mid- to late adolescence, a period marked by neurobiological maturation, social role transitions, and increasing academic and interpersonal demands [2–4,37].

Sex differences were pronounced, with female adolescents showing substantially higher prevalence of elevated mental health risk than male adolescents. This pattern is consistent with previous epidemiological and meta-analytic evidence showing higher levels of depressive symptoms and internalizing problems among adolescent girls [6,7]. These descriptive findings provide important epidemiological context for model interpretation, particularly because sex was the second most influential predictor after sadness/hopelessness in the primary SHAP analysis. However, these results should be interpreted as predictive and descriptive patterns rather than causal effects.

#### 4.1.2 Predictive capacity of the primary interpretable model.

The primary XGBoost model demonstrated good rank discrimination (AUC = 0.806; PR-AUC = 0.603). At the default threshold of 0.50, specificity was high (0.879) but sensitivity remained more modest (0.534). Importantly, the operating threshold was not selected using the held-out test data. Instead, five-fold out-of-fold predictions from the training sample were used to maximize the Youden index, yielding a locked threshold of 0.285. At this threshold, sensitivity increased to 0.754 while specificity remained 0.747, producing a balanced accuracy of 0.750. This demonstrates that much of the apparent sensitivity-specificity imbalance at threshold 0.50 reflected the arbitrary default cutoff rather than an inability of the model to distinguish higher- and lower-risk participants.

The resampling analyses supported this interpretation. Downsampling achieved sensitivity of 0.761 and specificity of 0.737, while the SMOTE-based model achieved sensitivity of 0.719 and specificity of 0.765. Because the threshold-calibrated primary model achieved similarly balanced performance without altering the class distribution, it was retained as the main model. The White-only internal validation also showed balanced performance (sensitivity = 0.757; specificity = 0.775; AUC = 0.825), suggesting that the improved case detection was not confined to the full mixed racial/ethnic sample.

Although the calibrated model achieved sensitivity and specificity above 0.700, it should not be interpreted as a ready-to-deploy clinical screening or diagnostic instrument. The selected threshold was optimized internally and would require recalibration and external validation before use in another population. The PPV at the locked threshold was 0.536, meaning that increased case detection came with a substantial number of false positives, whereas the NPV was high at 0.886. In a school or public health setting, such an operating point may be useful for auxiliary risk stratification or prioritizing supportive follow-up, but it should not replace direct self-report, clinical assessment, or established referral procedures.

### 4.2 Emotional, demographic, and behavioral predictors in the primary model

#### 4.2.1 Leading predictors and the role of sadness/hopelessness.

Sadness/hopelessness was by far the strongest predictor in the primary SHAP analysis. This is substantively plausible because Q25 captures a significant episode of sadness or hopelessness during the past 12 months, whereas the outcome Q85 captures whether mental health was not good most of the time or always during the past 30 days. The two variables are distinct YRBS items and Q25 does not directly define Q85; however, they are conceptually close and their reference periods can overlap. The strong SHAP contribution should therefore be interpreted as evidence that a proximal emotional-health indicator carries substantial information about current poor mental health, not as evidence of an independent causal effect.

The no-Sadness sensitivity analysis helps quantify this dependence. Excluding sadness/hopelessness reduced AUC from 0.806 to 0.736 and PR-AUC from 0.603 to 0.510, and sensitivity at the default threshold fell from 0.534 to 0.238. Nevertheless, the no-Sadness model retained moderate discrimination, indicating that demographic and behavioral factors still contained predictive information. Within the primary model, sex was the second most influential predictor, followed by sleep duration and screen time. Longer sleep generally contributed to lower predicted risk, whereas higher screen time contributed to higher predicted risk, consistent with prior evidence linking insufficient sleep and excessive screen exposure to poorer adolescent mental health [11–15].

#### 4.2.2 Modifiable behavioral factors.

Physical activity and breakfast frequency showed smaller but interpretable contributions in the primary model. More MVPA days generally contributed to lower predicted risk, consistent with evidence that physical activity is associated with better psychological well-being in youth [16–18]. Higher breakfast frequency also showed a generally protective predictive pattern, although its contribution was substantially smaller than that of sadness/hopelessness, sex, sleep duration, and screen time.

Dietary indicators such as fruit consumption, green salad intake, potato consumption, and carrot intake showed comparatively small global SHAP contributions. This does not imply that diet is unimportant for adolescent mental health; rather, the specific dietary indicators available in the YRBS contributed less predictive information than the proximal emotional indicator, sex, sleep duration, screen time, and breakfast frequency in this model. Prior studies have linked overall dietary quality to depression and anxiety through multiple potential pathways [19–21].

Taken together, the revised findings indicate that adolescent mental health risk stratification in this dataset is driven most strongly by a proximal emotional-health indicator, with additional information contributed by demographic characteristics and health behaviors. This distinction is important when considering whether the model is intended for explanatory research, population-level risk stratification, or practical screening.

### 4.3 Interpretable predictor patterns and unsupervised structure

The SHAP beeswarm, predictor distribution plots, and supplementary dependence plots showed interpretable directional patterns. Not reporting the 12-month sadness/hopelessness experience was associated with a large reduction in predicted risk, while the presence of that experience corresponded to substantially higher predicted risk. Longer sleep and more physical activity generally contributed toward lower predicted risk, whereas higher screen time contributed toward higher predicted risk. These behavioral patterns are consistent with evidence relating movement behaviors and sleep to adolescent mental health [18,22,38].

PCA provided complementary unsupervised context. The first two principal components explained only 18.28% of total variance and did not clearly separate adolescents with and without elevated mental health risk. PCA plots colored by sex and age group also showed substantial overlap. Thus, the supervised model's performance was not explained by a simple two-dimensional clustering structure, even though sadness/hopelessness was highly influential in the XGBoost model. This supports interpreting the model as combining information across several dimensions rather than recovering a single obvious latent split.

These patterns should not be interpreted as causal effects. The YRBS data are cross-sectional and self-reported, and the 12-month sadness/hopelessness period can overlap with the 30-day current mental health outcome period. SHAP values explain how variables contribute to model predictions within this dataset; they do not establish temporal precedence or etiological mechanisms.

### 4.4 Methodological contribution and public health implications

A key methodological lesson from this revision is the importance of verifying the exact questionnaire definitions underlying model variables. The 12-month sadness/hopelessness item and the 30-day current mental health item are related but distinct YRBS measures. Treating the former as a legitimate predictor produces substantially stronger discrimination, while the no-Sadness sensitivity analysis transparently shows how much predictive performance depends on this proximal emotional indicator. This dual analysis is more informative than treating the two items as identical or interpreting the primary model performance without considering their conceptual proximity.

The benchmark analyses further clarify the role of XGBoost. Logistic regression and the linear probability model achieved AUCs almost identical to that of XGBoost, while random forest was modestly lower. Therefore, the present results do not show a large predictive advantage for XGBoost over simpler models. Its main contribution is the SHAP-based characterization of feature importance and non-linear predictor patterns. Cross-demographic transfer analyses also showed that AUC remained moderate to good across racial/ethnic and sex transfer settings, but the balance between sensitivity and specificity varied substantially. For example, training on non-White participants and testing on White participants produced high specificity but low sensitivity, whereas male-to-female transfer produced high sensitivity but lower specificity. These results reinforce the need for subgroup-specific calibration rather than assuming a single operating threshold will transport uniformly across populations.

From a public health perspective, the primary model may support auxiliary risk stratification, but its practical meaning should be interpreted carefully. Because sadness/hopelessness is the dominant predictor, a high model score partly reflects an adolescent already reporting a substantial emotional symptom during the previous 12 months. The model therefore should not be presented as replacing direct questions about emotional distress. Instead, after external validation, it could integrate that information with sleep, screen time, physical activity, breakfast frequency, and demographic factors to prioritize supportive wellness follow-up or population-level prevention planning. Students reporting current emotional distress, self-harm, or other urgent concerns should receive direct professional assessment regardless of model score [39,40].

Future research should prioritize longitudinal prediction and external validation in independent samples, with thresholds recalibrated for the intended setting. Models that deliberately exclude proximal emotional-health indicators may also be useful when the goal is earlier identification based primarily on behavioral and contextual information, although the present no-Sadness analysis indicates that such models may have lower discrimination. Future work could also incorporate richer contextual, digital behavioral, or biological markers and assess calibration and fairness across demographic groups [41].

### 4.5 Strengths and limitations

This study has several strengths. It used a large national adolescent sample, applied an interpretable machine learning framework, separated the primary modeling and held-out test data, and selected the operating threshold using out-of-fold predictions within the training set rather than optimizing it on the test set. The analysis also verified the distinction between the Q25 sadness/hopelessness predictor and Q85 current mental health outcome, included a no-Sadness sensitivity analysis to quantify dependence on the proximal emotional predictor, compared XGBoost with multiple benchmark models, and examined internal subgroup and cross-demographic portability.

Several limitations should be acknowledged. First, the cross-sectional design precludes causal or prospective interpretation. Second, all measures were self-reported and may be affected by recall and social desirability bias. Third, sadness/hopelessness was the dominant predictor, and its 12-month reference period can overlap with the 30-day outcome period. Although it is a distinct item rather than the outcome itself, this conceptual and temporal proximity likely contributes substantially to model performance; the reduction to AUC 0.736 in the no-Sadness sensitivity analysis illustrates this dependence. Fourth, the threshold of 0.285 was selected internally and requires external recalibration and validation before practical use; its PPV of 0.536 also indicates a meaningful false-positive burden. Fifth, the reduced reproducible analytic dataset did not include YRBS survey weights, strata, or PSU variables, so descriptive proportions should not be interpreted as design-weighted national prevalence estimates. Sixth, cross-demographic transfer analyses showed uneven sensitivity-specificity balance across groups, indicating that subgroup-specific calibration may be needed. Seventh, the available behavioral and dietary indicators were relatively broad survey measures and may not capture the full complexity of adolescent lifestyle patterns. Finally, SHAP values explain model predictions but do not establish etiological mechanisms.

## 5 Conclusion

This study applied an interpretable machine learning framework to classify elevated current mental health risk among U.S. adolescents. After verifying that the 12-month sadness/hopelessness item and the 30-day current mental health outcome are distinct YRBS measures, sadness/hopelessness was retained in the primary predictor set. The resulting XGBoost model achieved an AUC of 0.806, and a threshold selected using training-set out-of-fold predictions yielded sensitivity of 0.754 and specificity of 0.747 in the held-out test set. Sadness/hopelessness was the dominant predictor, followed by sex, sleep duration, screen time, and breakfast frequency. Excluding sadness/hopelessness reduced discrimination, demonstrating that the model's performance depends substantially on this proximal emotional indicator while also showing that demographic and behavioral variables retain moderate predictive value. XGBoost did not materially outperform simpler linear benchmarks in AUC, but SHAP provided a transparent view of predictor contributions and non-linear patterns. The model may support auxiliary school-based or population-level risk stratification after external validation and threshold recalibration, but it should not replace direct self-report or professional mental health assessment.

## Supporting information

S1 Data Analytic dataset used for the reproducible analysis, provided as a compressed comma-separated text file. (GZ)

S1 File Reproducible R code for data preprocessing, model development and evaluation, sensitivity analyses, SHAP interpretation, and figure generation. (R)

S1 Fig SHAP dependence plots for the top predictors in the primary XGBoost model. (PNG)

S2 Fig Principal component analysis of the primary predictor set by sex. (PNG)

S3 Fig Principal component analysis of the primary predictor set by age group. (PNG)
